# Aluminum-based salts: a novel and simple way to treat molluscum contagiosum and cutaneous warts

**DOI:** 10.12688/f1000research.173851.1

**Published:** 2025-12-01

**Authors:** Jose F. Camargo

**Affiliations:** 1 Division of Infectious Diseases, University of Miami Department of Medicine, Miami, Florida, 33136, USA

**Keywords:** aluminum, molluscum contagiosum, human papillomavirus, verruca vulgaris, warts

## Abstract

Molluscum contagiosum and human papillomavirus (HPV) are very common cutaneous infections with worldwide distribution, yet treatment options remain limited. Traditional therapies are invasive and have a high rate of recurrence, and newer topical agents available are costly.

Here I discuss evidence supporting the hypothesis that the aluminum-based salts, utilized in the antiperspirant industry since the early 1900s, could have a therapeutic effect on skin lesions due to molluscum contagiosum, HPV and possibly other viruses. Clinical trials in this area are needed.

Cutaneous human papillomavirus (HPV) infections are ubiquitous in the general population. Nearly everyone will get HPV at some point in their lives. Up to 90% of healthy individuals test positive for beta HPV types. ^1^ Numerous treatments for nongenital cutaneous warts are available, although no single therapy has been established as completely curative; this is portrayed by the fact that even duct tape is considered a treatment option. ^2^ Molluscum contagiosum (MC) is another cutaneous viral infection with prevalence in the U.S. general population of approximately 5%, affecting primarily pediatric patients, sexually active young adults, and immunocompromised people of all ages. ^3^ Spontaneous resolution can take months to years impacting the quality of life of patients and caregivers. Traditional therapies are invasive and have a high rate of recurrence. In the last two years, however, the Food and Drug Administration (FDA) approved two topical agents to treat MC. The efficacy difference of the study drugs compared to the vehicle (placebo) in the trials was 28-41% for cantharidin, ^4^ and 13% for berdazimer gel. ^5^ Immunocompromised hosts were excluded from these clinical trials and no data on recurrence was reported. Notably, both agents are considerably expensive and, in some cases, must be given for up to 12 weeks.

I am now an infectious diseases specialist with over a decade of experience, but 25 years ago, while still in my third year of medical school, I noticed that my youngest brother, age 11 at the time, had 2 small non-painful non-itchy pearly skin lesions right below his armpit at the level the midaxillary line. I thought it was a case of MC; I reassured him and explained it would probably go away within a few months. Around the same time, he began experiencing puberty sweat and he did not hesitate in using my roll-on deodorant (I cannot remember the brand, but I remember its green pine color). Within days of using deodorant, I was amazed to notice how his skin lesions rapidly cleared up. Last year, my 10-year-old daughter had a 1 cm rough-surface wart at the lateral aspect of the 4 ^th^ distal interphalangeal joint that had been present for months. Without pursuing draconian IRB proceedings and without much published scientific literature to support my hypothesis, I started applying deodorant gel (active ingredient: aluminum zirconium octachlorohydrex Gly 16%; inactive ingredients: water, denatured alcohol, cyclopentasiloxane, propylene glycol, dimethicone, calcium chloride, PEG/PPG-18/18 dimethicone, fragrance) once or twice a day to the lesion. Within less than two weeks, the wart was notably better in size and height, and within four weeks it had nearly resolved. More recently, encouraged by these results, I applied the same deodorant gel, once a day, to a small (2-3 mm) dome-shaped pearly papule in the right nasolabial fold of my 7-year-old daughter, which I thought was MC; within 5-6 applications, the lesion completely disappeared. I am now convinced that the antiperspirant salts shortened the duration of symptoms in these three cases.

Although the terms antiperspirant and deodorant are often used interchangeably, they are not synonyms. Deodorants target bacteria that create odors while antiperspirants contain aluminum-based salts that form a colloidal plug preventing, temporarily, the eccrine glands from releasing sweat. ^6, 7^ In addition to the anecdotal evidence mentioned above, the following observations provide circumstantial evidence supporting the possibility that the aluminum-based salts, utilized in the antiperspirant industry since the early 1900s, ^6, 7^ could have a beneficial effect on skin lesions due to MC, HPV and possibly other viruses:

1. Today over 90% of the U.S. population uses some form of underarm antiperspirant daily; ^7^ and although it has been reported, HPV associated warts involving the armpit are an extremely rare occurrence. ^8, 9^
2. Aluminum salts (administered by mouth) shortened the duration of symptoms and had beneficial effects on laboratory findings in patients with COVID-19. ^10^
3. In Turkey, animals suffering from foot and mouth disease are treated, presumably quite successfully, with the topical use of aluminum salts; ^10^ and in animal models, intranasal administration of aluminum-based adjuvants alone can prevent influenza or respiratory syncytial virus by activating the alveolar macrophage and type I interferon pathway components of the innate immune system. ^11^
4. Aluminum (Al2O3), zirconium (ZrO2), and cerium (CeO2) oxides nanoparticles exhibit high affinity for human-pathogenic viruses ^12^ such as adenovirus and human immunodeficiency virus type 1; and can sequester viral nucleic acids from aqueous solutions. ^12, 13^ Al2O3 nanoparticles have a robust antiviral activity against herpes simplex virus-1 *in vitro.* ^14^ In addition, inorganic nanoparticles may also exert direct virucidal activity through various mechanisms. ^15^
5. Aluminum-based salts have been proven to enhance the immune response. Since the 1930s aluminum-based salts (hydroxide or phosphate) have been used as adjuvants for numerous human vaccines, ^16^ including the HPV vaccine (Gardisil 9). AS04 is a combination of the toll-like receptor 4 agonist monophosphoryl lipid A (MPL) and aluminum hydroxide. The AS04-adjuvanted HPV vaccine induces a high and sustained immune response against HPV. ^17^ Other examples of aluminum-containing vaccines are listed in the U.S. Centers for Disease Control and Prevention (CDC) website, and include DTaP (Daptacel), DTaP (Infanrix), DTaP-HepB-IPV (Pediarix), DTaP-IPV (Kinrix), DTaP-IPV (Quadracel), DTaP –IPV/Hib (Pentacel), DTaP-IPV-Hib-HepB (VAXELIS), HepA (Havrix), HepA (Vaqta), HepB (Engerix-B), HepB (Recombivax), HepA/HepB (Twinrix), HIB (PedvaxHIB), Japanese encephalitis (Ixiaro), MenB (Bexsero, Trumenba), Pneumococcal (Prevnar 13, Prevnar 20, VAXNEUVANCE), Td (Tenivac), Tdap (Adacel), Tdap (Boostrix), and Tick-Borne Encephalitis (TICOVAC). Aluminum enhances antigen uptake by antigen-presenting cells and stimulates components of the inflammasome. ^16, 18, 19^ Aluminum hydroxide also prolongs the cytokine responses at the injection site and induces chemokine (namely CCL2) production. ^16, 18, 19^ These effects of aluminum salt have been linked to monocyte differentiation and migration to draining lymph nodes and augmented humoral response. ^16, 18, 19^
6. Aluminum-based salts have been used with promising results in small case series of verruca vulgaris. Alum (potassium aluminum sulfate) solution was effective in the treatment of common warts in two patients from Iran. ^20^ In a more recent report from Turkey, four patients with cryotherapy resistant HPV warts were successfully treated with an 18% aluminum chloride solution. ^21^ The authors proposed that aluminum chloride solution acted as an astringent with keratolytic effect in the virus-infected epidermis.

But are aluminum salts safe? Aluminum salts are common in nature and are also found in drinking water in trace amounts; and have been used for many years as ingredients in drugs, natural health products and cosmetics. ^10^ It is estimated that only 0.0005 to 0.012% of applied aluminum in an antiperspirant formulation is absorbed through the skin. ^22, 23^ In addition, aluminum-containing adjuvants have been used for nearly a century in vaccines injected into more than 1 billion people worldwide without safety concerns. ^16^

Some of the paramount advancements in medicine have been the result of basic clinical observations. In 1796, Edward Jenner, considered the father of immunology, inoculated an 8-year-old with matter from a cowpox sore on the hand of a local milkmaid, merely based on tales of dairymaids being naturally protected from smallpox after cowpox. The child did not only experience a full recovery but also became immune to smallpox. ^24^ Smallpox would become the first and only infectious disease eradicated through mass immunization.

No, I do not believe that my observations are as important as those from the times of Jenner, nor that Windex has healing properties, ^25^ but I do strongly believe that aluminum-based salts might have salutatory effects on cutaneous viral infections. Serendipity has played a role in some of the most important medical discoveries in the last century. ^26^ Penicillin was discovered by accident; ^26^ remdesivir, the first FDA-approved antiviral for COVID-19, was originally developed for the treatment of Ebola virus; ^27^ sildenafil (Viagra) was initially developed as an anti-angina medication; ^26^ warfarin was initially developed as rat poison; ^26^ and among these lines, it might just be that an inexpensive, effective (conceivable better than duct tape) and well tolerated topical treatment for MC or cutaneous warts has been hiding in your bathroom cabinet all along. The role of aluminum-based salts in the treatment of MC, HPV and other skin viral infections deserves further study.

## Data Availability

No data associated with this article.
