# Supplementary material for: Aluminum-based salts: an unorthodox but simple way to treat molluscum contagiosum and cutaneous warts
Source: F1000Res. 2026 Jul 13;14:1340. Originally published 2025 Dec 1. [Version 2] doi: 10.12688/f1000research.173851.2 (PMC13541097; doi:10.12688/f1000research.173851.2)
Supplement: Peer review history

# Reviewer report: Reviewer response for version 2

**DOI:** 10.5256/f1000research.205829.r510583

**Published:** 2026-09-03

**Authors:** Aurora Marques Cianciarullo

**Affiliations:** 1 Laboratory of Genetics, Butantan Institute, Secretary of Sao Paulo State for Health, Sao Paulo, Brazil

The article's title is quite appropriate. The text is very well written and impresses with its insight, dating back several years, in correlating the use of antiperspirants with the reduction and disappearance of warts in family members. Antiperspirants contain aluminum-based salts, not present in deodorants, which form a colloidal plug, temporarily preventing the secretion of sweat by the eccrine glands, as mentioned by the author.

 I fully agree that a formal clinical trial should be initiated, meeting the requirements of regulatory agencies, considering the evidence obtained from existing case reports in the literature. Once it is confirmed that aluminum salts have the property of interacting with warts of viral origin and eliminating them, other dermatitis could be investigated simultaneously and treated, such as seborrheic dermatitis, a chronic inflammation without a definitive cure, as this would bring great benefit to those suffering from all these skin diseases.

  Another interesting approach to the topic would be to research the mechanisms involved in this therapeutic process using aluminum salts.

 Congratulations!

Is the topic of the opinion article discussed accurately in the context of the current literature?

Yes

Are arguments sufficiently supported by evidence from the published literature?

Yes

Are all factual statements correct and adequately supported by citations?

Yes

Are the conclusions drawn balanced and justified on the basis of the presented arguments?

Yes

Reviewer Expertise:

Cell Biology, Microbiology, Viral Biotechnology, Vaccines.

I confirm that I have read this submission and believe that I have an appropriate level of expertise to confirm that it is of an acceptable scientific standard.

# Reviewer report: Reviewer response for version 1

**DOI:** 10.5256/f1000research.191704.r452166

**Published:** 2026-01-29

**Authors:** Maher Al-Muriesh, Bilal Abdul fattah

**Affiliations:** 1 Sana’a University, Sana’a, Yemen; 2 Chtoura Hospital, chtoura, Lebanon

The manuscript is well written

 some suggestions :

 Aluminum-based salts were used previously in the literature to treat cutaneous warts. Therefore, in this manuscript, delete 'cutaneous wart' from the title if you want to keep the word ( novel).

 Because the manuscript is an opinion article, it is not mandatory to add images of the case, although adding before-and-after treatment figures would strengthen the article.

Is the topic of the opinion article discussed accurately in the context of the current literature?

Partly

Are arguments sufficiently supported by evidence from the published literature?

Yes

Are all factual statements correct and adequately supported by citations?

Yes

Are the conclusions drawn balanced and justified on the basis of the presented arguments?

Yes

Reviewer Expertise:

dermatology

We confirm that we have read this submission and believe that we have an appropriate level of expertise to confirm that it is of an acceptable scientific standard.

# Response

**Published:** 2026-01-29

**Authors:** Jose Camargo

**Affiliations:** University of Miami Miller School of Medicine, USA

I thank the reviewer for his insightful comments and suggestions. I agree with the first suggestion, and the title will be edited accordingly (only waiting for additional reports before starting on any article revisions). Unfortunately, since these anecdotal observations occurred in a personal -not hospital- setting, no pictures are available.

# Response

**Published:** 2026-07-07

**Authors:** Jose Camargo

**Affiliations:** University of Miami Miller School of Medicine, USA

Tittle has been edited following the reviewer's recommendation.
